# Blood pressure management in secondary prevention after myocardial infarction

**DOI:** 10.1093/cvr/cvaf145

**Published:** 2025-08-26

**Authors:** Massimo Volpe, Giovanna Gallo, Tomasz J Guzik, Bryan Williams, Franz H Messerli

**Affiliations:** Department of Clinical and Molecular Medicine, Sapienza University of Rome, Via di Grottarossa 1035-1035, 00189 Rome, Italy; IRCCS S. Raffaele, Via della Pisana 235, 00163 Rome, Italy; Department of Clinical and Molecular Medicine, Sapienza University of Rome, Via di Grottarossa 1035-1035, 00189 Rome, Italy; Cardiology Unit, Sant'Andrea University Hospital, Rome, Italy; Centre for Cardiovascular Sciences, BHF Cardiovascular Research Centre, University of Edinburgh, Edinburgh, UK; Department of Medicine, Jagiellonian University, Krakow, Poland; UCL Hospitals Biomedical Research Centre, University College London (UCL), Institute of Cardiovascular Science, National Institute for Health Research, London, UK; Kantonsspital Luzern and Department of BioMedical Research, University of Bern, Bern, Switzerland

**Keywords:** Blood pressure management, Secondary prevention, Acute coronary syndromes, Coronary artery disease, J-curve phenomenon

## Abstract

The management of blood pressure (BP) in secondary prevention after acute myocardial infarction (AMI) remains a matter of debate. Since no dedicated trials have specifically addressed BP control in patients recovering from an acute coronary syndrome, there are no evidence-based, prospective data to define precise BP targets in this population. Moreover, major international guidelines devote surprisingly little attention to BP management in the post-AMI setting, despite its recognized importance in reducing recurrent cardiovascular events. On one hand, BP-lowering may improve cardiac function by reducing afterload and myocardial oxygen consumption and by facilitating favourable ventricular remodelling. On the other hand, the concept of a J-shaped association—whereby excessive lowering of diastolic blood pressure may paradoxically increase cardiovascular risk, particularly in the early post-AMI period—remains a matter of ongoing uncertainty. Despite decades of investigation, this issue has not been definitively resolved and continues to raise debate within both the scientific community and among clinicians. In this review, we examine the current evidence supporting BP control in the context of secondary prevention following AMI, with an updated focus on the ongoing debate surrounding the potential implications of the J-shaped phenomenon.

## Graphical Abstract

**Graphical Abstract:**
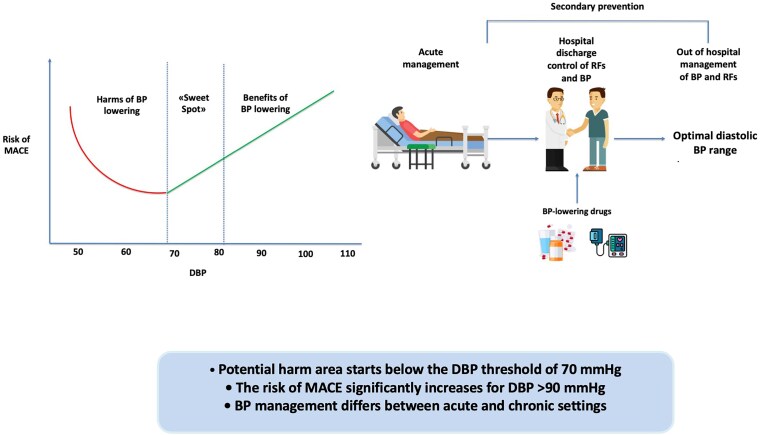
BP levels and cardiovascular risk in AMI secondary prevention. AMI, acute myocardial infarction; BP, blood pressure; DBP, diastolic blood pressure; MACE, major cardiovascular events; RFs, risk factors.

## 1. Introduction

The management of blood pressure (BP) after acute myocardial infarction (AMI) remains a topic of debate. The 2018 European Society of Cardiology (ESC)/European Society of Hypertension (ESH) guidelines were the first to recommend lower BP targets (systolic blood pressure [SBP] between 120 and 129 mmHg and diastolic blood pressure [DBP] between 70 and 79 mmHg) for most hypertensive patients aged 18–65 years, including those with prior coronary and cerebrovascular events.^1,2^ These recommendations were reaffirmed in the 2023 ESH guidelines for patients with a history of coronary artery disease (CAD).^3^ BP-lowering drugs, such as renin-angiotensin system inhibitors (RASi), beta-blockers (BB), and calcium channel blockers (CCB), are integral to CAD management, regardless of their antihypertensive effects. Therefore, the challenge is not whether to prescribe these medications post-AMI, but rather how to optimize drug selection and dosing to meet recommended BP targets.^3^ The 2024 ESC guidelines also endorse these BP goals and discourage de-escalating treatment in asymptomatic patients with on-treatment BP <120/70 mmHg. They further emphasize using BP-lowering medications based on their established benefits in CAD management.^4^ Caution is recommended in older patients (aged ≥85 years), or with moderate-to-severe frailty, orthostatic hypotension, and limited life expectancy (<3 years). In these categories, it is also suggested to defer the prescription of an antihypertensive treatment until BP is >140/90 mmHg, since the net benefit from BP-lowering drug therapy remains unclear.^4^

Similarly, American Heart Association (AHA)/American College of Cardiology (ACC) guidelines recommend a BP target of <130/80 mmHg in patients with CAD and advocate initiating treatment at BP levels ≥130/80 mmHg.^5^ Canadian guidelines recommend starting a pharmacological treatment for adults with BP ≥140/90 mmHg and for those with SBP between 130 and 139 mmHg at high cardiovascular risk. It is recommended to achieve a target SBP <130 mmHg, provided the treatment is well tolerated.^6^

Despite the emphasis on BP control in secondary prevention, the scientific literature supporting these recommendations remains relatively sparse. Specific trials dedicated to BP management in secondary prevention after AMI are lacking, and definitive BP targets based on prospective evidence have yet to be established. On one hand, BP reduction can be beneficial by decreasing cardiac afterload, reducing myocardial oxygen consumption, and promoting favourable ventricular remodelling, thereby enhancing cardiac function.^7^ On the other hand, concerns persist regarding the J-shaped curve phenomenon, which suggests that excessive DBP lowering may increase cardiovascular risk.^8^ In such a context, the balance of potential harm vs. benefit (‘primum non nocere’) is a fundamental principle in medicine and aggressive lowering BP, is often viewed with concern in high-risk conditions, such as in post-AMI patients.

This review explores the available evidence supporting current BP targets in secondary prevention after AMI, and also provides an updated focus on the controversial role of the J-curve phenomenon (*Graphical abstract*).

## 2. J-curve phenomenon: the ‘dark side of the moon’ of the antihypertensive treatment after acute myocardial infarction

The concept of a J-curve phenomenon—where cardiovascular risk increases at both high and low extremes of BP—was first introduced over 40 years ago.^9^ Since then, numerous studies have attempted to define a lower BP threshold beyond which cardiovascular risk post-AMI may rise, particularly for DBP. *Table 1* summarizes the main features of the most relevant trials exploring this aspect in the setting of secondary prevention.^10–20^ Most studies suggest that lowering DBP below 60–80 mmHg offers no additional benefit and may even be harmful. However, heterogeneity in study design, populations, and therapeutic interventions contributes to uncertainty regarding the precise threshold of risk. A potential role of an extreme DBP reduction (<60 mmHg) in increasing the risk of major cardiovascular events (MACEs) has been reported also in studies that included patients with high cardiovascular risk but without previous cardiovascular events (*Table 2*).^21–31^ However, it should be underlined that most of these studies were designed to investigate the role of SBP lowering, and in some cases (e.g. SPRINT) the intensive treatment group was aimed to achieve an SBP target <120 mmHg.

**Table 1:** Summary of the studies investigating the potential role of a J-curve phenomenon in the BP management after myocardial infarction

Study	Methodology	Key Findings
Anderson *et al*.^10^	Data from the Framingham study	Lack of benefits in patients achieving DBP <90 mmHg
Cruickshank *et al*.^11^	902 patients with CAD	Increased incidence of MI for DBP <85–90 mmHg.
Farnett *et al.*^12^	Meta-analysis of 13 studies, 48 000 individuals	Relationship between DBP <85 mmHg and cardiac events; no association with stroke.
Hypertension optimal treatment (HOT)^13^	18 790 patients randomized to DBP goal ≤90, ≤85, and ≤80 mmHg	No significant differences in MACE, J-curve relationship for MI in CAD patients.
Framingham offspring study^14^	791 individuals post-cardiovascular event	Five-fold higher recurrent MACE risk with DBP <70 mmHg
INVEST study^15^	22 576 hypertensive patients with CAD	J-curve effect present, lost in revascularized group.
EPHESUS study^16^	6642 MI and HF patients	DBP <70 mmHg increased CV death or hospitalization risk in non-reperfused patients.
Kaiser Permanente study (KPNC)^17^	1.3 million individuals	J-curve relationship detected, but significance lost after adjustments.
CLARIFY study^18^	22 672 adults with stable CAD	J-shaped effect observed for SBP and DBP; DBP <60 mmHg increased CV death and MI risk.
TNT trial^19^	10 001 patients with CAD	J-curve noted for both SBP and DBP with nadir at 146.3/81.4 mmHg.
PROVE IT-TIMI 22 Trial^20^	4162 ACS patients	J-shaped relationship between BP and both primary and secondary outcomes.

**Table 2:** Summary of the studies investigating the potential role of a J-curve phenomenon in the BP management in high-risk coronary artery disease

Study	Methodology	Key findings
ONTARGET^21^/TRANSCEND^22^ Trials^23,24^	Combined population of over 30 000 patients	DBP <70 mmHg associated with increased MI, HF, and mortality risk.
ARIC Study^25^	11 565 adults without HF and previous MACE	DBP <60 mmHg associated with increased CAD and mortality but not stroke.
MESA Study^26^	6811 participants without known CVD	DBP <60 mmHg linked to coronary events and all-cause mortality
SAVOR-TIMI 53 Trial^27^	16 492 patients with diabetes mellitus	DBP <60 mmHg linked to higher cardiovascular death, MI, and stroke rates.
Del Pinto *et al*. SPRINT post-hoc analysis^28^	9361 high CV risk patients	Higher risk of cardiovascular events with DBP <60 mmHg
Khan et al. SPRINT post-hoc analysis^29^	1519 participants with and 7574 without prior cardiovascular disease	Significant J-shaped relationship between mean on-treatment DBP and the primary outcome independently from the history of cardiovascular disease
Lee *et al*. SPRINT post-hoc analysis^30^	8046 with a baseline DBP ≥65 mmHg	Diastolic hypotension <55 mmHg had an increased risk for MI, other acute coronary syndromes, stroke, HF, and all-cause death.
Li *et al*.^31^	Combined analysis of SPRINT and ACCORD-BP data	DBP <60 mmHg significantly increased risks of death, MI, and stroke

When we applied an artificial intelligence (AI) platform-based analysis to deeply explore the results of the available studies,^32^ a J-curve phenomenon was revealed as plausible for levels of DBP <65 mmHg.^32^ Stratifying the data for the presence of CAD or for age ≥65 years, DBP values <70 mmHg appear to be associated with an increased risk of MACEs. In these categories of patients, the AI optimal DBP target falls in the range of 70–80 mmHg. On the other hand, in subjects without a history of coronary events or aged <65 years, a raised risk of MACEs is reported only for DBP <60 mmHg, thus suggesting the safety of tighter therapeutic targets in these groups, with a proposed optimal DBP goal ranging between 75 and 65 mmHg.

### 2.1 Pathophysiological mechanisms

#### 2.1.1 Myocardial oxygen supply/demand ratio and perfusion pressure (PP)

Unlike in other organs, including the brain and kidneys, myocardial perfusion and oxygen delivery through coronary flow mostly occur during diastole, accounting for about 80% of the total coronary blood supply.^11,33–35^ In the heart, the compression of the vasculature by its surrounding muscle during systole decreases flow.^36^ Thus, a simple pressure gradient model in which flow moves from the highest to the lowest pressure does not fully explain myocardial flow. Blood is primarily driven into the epicardial coronaries by a forward-moving pushing wave generated during systole, and then it is released for forward flow when the myocardium relaxes during diastole. A second larger wave is generated by the relaxation of the left ventricle (LV) with a suction mechanism.^36^ Coronary PP (CPP) reflects the gradient between the DBP and the LV diastolic pressure and when it is lowered to 40–50 mmHg, the so-called pressure at zero flow, diastolic blood flow in the coronary arteries ceases. The CPP decrease may be counter-regulated by coronary autoregulation, a metabolic microvascular vasodilatation that can produce a five-fold increase in blood flow, ensuring relatively constant myocardial perfusion over a wide CPP range of 45–125 mmHg.^11,33–36^

#### 2.1.2 Coronary artery disease, LV hypertrophy (LVH)

In the presence of coronary artery stenosis, the coronary capacity to increase blood flow is exhausted, resulting in a reduced CPP. Indeed, the presence of occlusive CAD shifts the lower autoregulatory limit upward. This phenomenon may be further exacerbated when hypertension coexists, particularly in the presence of vascular remodelling and LVH. Indeed, aortic stiffness may contribute to reducing DBP due to an impaired reservoir function of the aorta.^37^ In addition, LVH is associated with an increase in LV diastolic pressure, thus maximal coronary flow can increase only by three-fold, with a subsequent drop in effective myocardial perfusion pressure, even though the oxygen request is augmented, generating a condition of ischaemia especially in the sub-endocardium.^11,33–36^

#### 2.1.3 Coronary microvascular remodelling

A concentric coronary microvascular remodelling occurs in LVH, with a reduction of microcirculatory compliance and of maximum myocardial blood flow even in the absence of epicardial stenoses, leading to the need of a higher driving pressure to maintain coronary perfusion in the setting of higher heart rates and O2 demand.^38^ Accordingly, an impairment in coronary flow reserve (CFR) has been demonstrated in hypertensive subjects. CFR alteration has been identified as a determinant of myocardial diastolic dysfunction and contributes to coronary flow reduction.^39^

It has been also demonstrated that in hypertensive patients CFR is transmurally blunted due to a reduced hyperaemic response to stress, also in the absence of coronary stenosis, this phenomenon being the expression of severe coronary microvascular dysfunction.^40^ In this regard, a large meta-analysis including 59 740 individuals has shown that abnormal CFR is associated with a higher incidence of MACE in patients with acute coronary syndromes (ACS) (hazard ratio [HR], 3.76; 95% confidence interval [CI], 2.35–6.00).^41^

#### 2.1.4 Vascular stiffness, loss of Windkessel effect

Lower DBP may be the expression of a stiff arterial tree and of an impairment of aortic reservoir,^37^ as it is observed in the elderly and in the presence of a remarkable systemic atherosclerotic burden.

A strong significant association has been demonstrated between DBP levels and coronary artery atherosclerosis quantified by the SYNTAX Score and SYNTAX Score II in stable patients with obstructive CAD, potentially contributing to explaining the correlation with a poor prognosis.^42^ In a cohort of 10 876 patients undergoing percutaneous coronary intervention (PCI), SBP >120 mmHg, and DBP <70 mmHg were associated with increased MI and mortality.^43^ The INdividual Data ANalysis of Antihypertensive intervention (INDIANA) registry^44^ and the CLARIFY study^23^ also demonstrate the persistence of the J-curve effect, even in patients with low PP ≤45 mmHg. These findings highlight the challenge of disentangling the effects of low DBP, wide PP, and aging in predicting MACE risk.

#### 2.1.5 Reverse causality

‘Reverse causality’ is another proposed mechanism for the J-curve effect, wherein low BP is a consequence of underlying frailty and comorbidities rather than a direct cause of increased risk.^45^ The worse outcomes in these patients with lower BP might be attributable not to a direct causative effect of treatment but to the effect of concomitant diseases causing a reduction in BP together with adverse outcome.^46,47^

### 2.2 Type-2 AMI vs. type-1 AMI

It has been also hypothesized that Type-2 AMI may be the predominant cause of the up-swinging limb of the J-curve with decreasing DBP. The pathophysiological mechanism of Type-2 AMI consists in a supply/demand mismatch, i.e. an imbalance in myocardial oxygen supply or an unmet increase in myocardial oxygen demand. Obviously, such a mismatch can occur with or without clinically significant CAD.^48^ Hence, hypotension or tachyarrhythmias are the most common causes of Type-2 AMI. In this clinical setting, only excessively high or excessively low BP levels are associated with a significant risk, particularly in older patients. Because mild hypertension is prone to mitigate the risk of hypotension, it may paradoxically exert a protective effect regarding the outcome of Type 2 AMI, as recently documented.^49^ A diagnosis of hypertension was found to be associated with lower all-cause mortality after Type-2 AMI.

**Figure 1:**
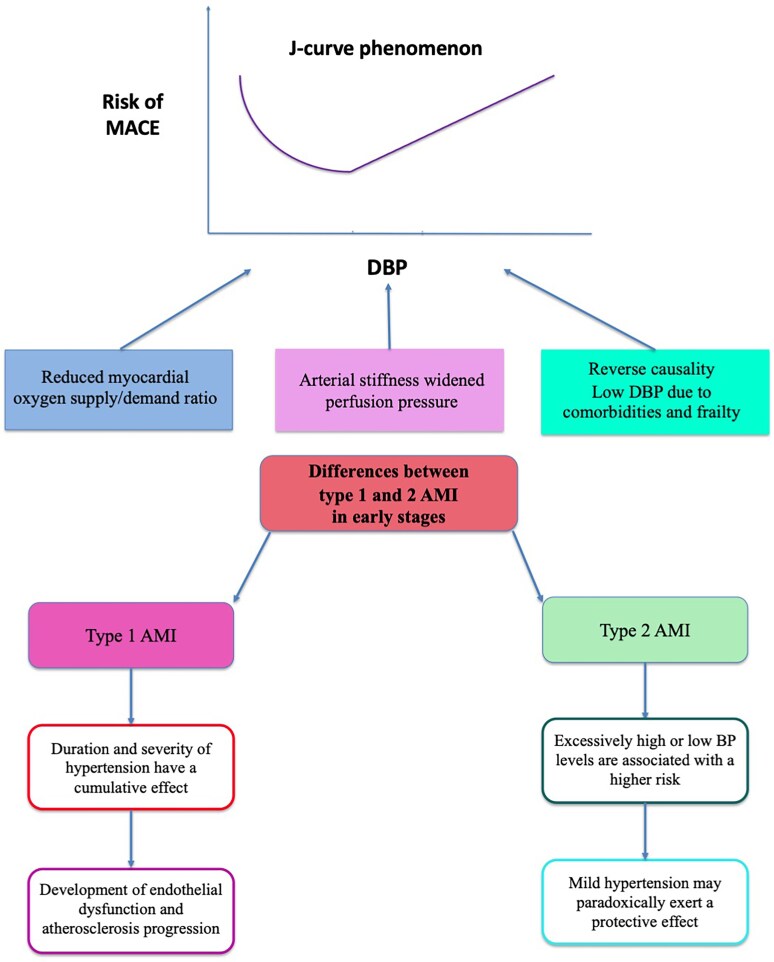
Differences in J-curve effect between Type 1 and 2 AMI. AMI, acute myocardial infarction; BP, blood pressure; DBP, diastolic blood pressure; MACE, major cardiovascular events.

Conversely, in Type-1 AMI, caused by acute atherosclerotic plaque rupture and coronary thrombosis, hypertension has been identified as one of the most important risk factors, due to its role in the development of endothelial dysfunction and atherosclerosis progression. In such a context, the duration and severity of hypertension have a cumulative effect, as it is reported for acute cerebrovascular events.^50,51^ (*Figure 1*).

Given the available evidence, the question is not whether the J-curve exists, but rather at what BP thresholds cardiovascular risk increases. Conceivably, there may also be target organ heterogeneity in that the nadir of the J-curve is different for the heart, the brain, and the kidney. The variability in study populations, treatment strategies, and follow-up durations complicates the definition of universal BP targets in post-MI. In particular, the role of DBP in contributing to a paradoxical rise in the risk of cardiovascular events most likely has a different impact in the early and late stages of MI. However, it remains unproven, and further studies specifically addressing the effects of different DBP targets in post-MI are needed.

## 3. Hypertension management after coronary artery events

Hypertension is a well-established risk factor for adverse outcomes post-MI. Multiple pathophysiological mechanisms, including endothelial dysfunction, LVH, sympatho-adrenergic and renin-angiotensin-aldosterone system (RAAS) activation, and increased oxidative stress, contribute to CAD progression.^52–54^ Moreover, long-standing and uncontrolled hypertension may enhance ischaemia and ischaemia-reperfusion injury mainly because of impaired microcirculation, increased vascular resistance, and reduced CFR.^55^ Recent registry data from ISACS-STEMI COVID-19, which included 16 083 STEMI patients undergoing PCI, revealed that those with a history of hypertension had significantly higher in-hospital (HR 1.673, 95% CI 1.389–2.014, *P* < 0.001) and 30-day mortality (HR 1.418, 95% CI 1.230–1.636, *P* < 0.001).^56^

In addition, although a large body of evidence has demonstrated that BP control is one of the most effective therapeutic strategies in the primary prevention of CAD, fewer data are available about the role of BP-lowering treatment in the recurrence of coronary events.^57^

BP control remains a cornerstone of secondary prevention. In the Korea Acute Myocardial Infarction Registry (KAMIR) registry (10 337 post-PCI AMI patients), the lowest MACE rates occurred at SBP 110–119 mmHg and DBP 70–79 mmHg.^58^

In pharmacological trials, the Comparison of Amlodipine Versus Enalapril to Limit Occurrences of Thrombosis (CAMELOT) study demonstrated that patients achieving BP <120/80 mmHg had regression of coronary atheroma volume, suggesting that more aggressive BP lowering may be beneficial in CAD.^59^ Altogether, these considerations should support ‘the lower the better’ strategy in secondary prevention in patients with a previous MI.

In PARADISE-MI, 5661 patients with MI complicated by LVEF, pulmonary congestion, or both were randomized to receive either sacubitril/valsartan (200 mg twice daily) or ramipril (5 mg twice daily) in addition to recommended therapy. Sacubitril/valsartan was not associated with a significantly lower incidence of death from cardiovascular causes or incident HF than ramipril.^60^ In both groups average baseline SBP was 121 mmHg. Although the overall incidence of adverse effects attributed to the therapies was similar in the two groups, more discontinuations attributed to hypotension were observed with sacubitril/valsartan.^60^

In the EMPACT-MI, 6522 patients who had been hospitalized for acute MI and were at risk for HF were randomized to receive empagliflozin at a dose of 10 mg daily or placebo in addition to standard treatment. Empagliflozin did not lead to a significantly lower risk of the composite of first hospitalization for HF or death from any cause as compared to placebo, although the incidence of HF was lower in the active group when the components of the endpoint were separately analysed.^61^

A meta-analysis investigated the benefits of antihypertensive medications in a population of 64 162 patients with history of CVD but without hypertension, showing a significant reduction of stroke [relative risk (RR) 0.77, 95% CI 0.61- 0.98, absolute risk], MI (RR 0.80, 95% CI 0.69–0.93), HF (RR 0.71, 95% CI 0.65–0.77), a composite MACE endpoint (RR 0.85, 95% CI 0.80–0.90), cardiovascular mortality (RR 0.83, 95% CI 0.69–0.99) and all-cause mortality (RR 0.87, 95% CI 0.80–0.95).^56^ The corresponding absolute risk reductions per 1000 persons were −7.7 for stroke, −13.3 for MI, −43.6 for chronic HF, −27.1 for composite MACE, −15.4 for cardiovascular mortality, and −13.7 for all-cause mortality. These results were independent from clinical history of MI or CAD and HF.^62^

Large-scale meta-analyses, such as the Blood Pressure Lowering Treatment Trialists’ Collaboration (BPLTTC) individual participant level data (IPD)study involving 344 716 participants, confirm that even modest BP reductions significantly reduce MACE risk, regardless of baseline BP or prior cardiovascular history.^63^ In the IPD meta-analysis of the BPLTTC a 5-mmHg reduction in SBP reduced the risk of MACE by about 10% even at normal BP levels in subjects with and without CVD. In this study, however, the effect was modelled according to 5-mmHg reductions, even though this was not achieved in some of the trials. In addition, it should be noted that the potential treatment harm was not investigated in this meta-analysis.^63^

**Figure 2:**
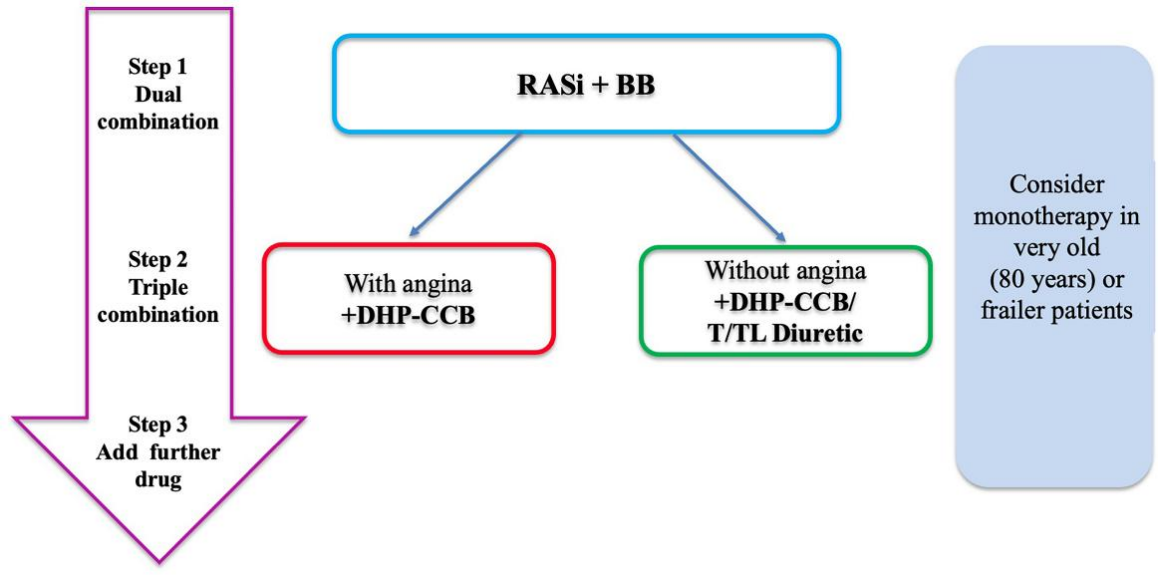
ESH guidelines therapeutic algorithm in hypertensive patients with CAD. BB, beta-blockers, CCB, calcium channel blockers; DHP, dyhidropiridinic; RASi, renin angiotensin system inhibitors; T, thiazide; TL, Thiazide-like.

With regard to the recommended therapeutic approach in patients with coronary disease, *Figure 2* reproduces the algorithm suggested by ESH guidelines.^3^ The same guidelines suggest that BP targets should be achieved within three months in all patients, including those with previous MI, independently from the chosen pharmacological strategy. Among the recommended BP-lowering strategies in patients with CAD, RAAS inhibitors, and CCB reduce LVH, improve arterial compliance, and decrease PP, due to a more pronounced effect on SBP than on DBP, while beta-blockers are associated with more prolonged diastolic phase and coronary perfusion time by decreasing heart rate.^36^Although several large randomized trials have provided evidence supporting the concept that an early BP control may produce long-lasting BP reductions and decrease the incidence of cardiovascular events also in patients with high cardiovascular risk (quote VALUE,^64^ ALLHAT,^65^ ASCOT-BPLA,^66^) precise and univocal data are lacking about the safety and the benefits of a fast achievement of therapeutic goals in secondary prevention settings and about the ideal time-frame to reach BP targets.^67^

It is also important to consider that the risk of recurrent cardiovascular events after MI might be largely influenced by the use of concomitant medications such as statins and antiplatelet drugs, which reduce the risk of future atherosclerotic events in patients in secondary prevention after ACS.^68,69^ For this reason, the effects of BP lowering in these patients must be assessed in the context of the overall therapeutic strategy. In this regard, a polypill containing different medications (statin, aspirin and ACE inhibitors) resulted in a lower risk of MACEs than a usual-care strategy of administration in older patients with recent myocardial infarction in the absence of substantial differences in BP and cholesterol levels, probably due to a greater therapeutic adherence.^70^

A final consideration is that the first 30 days post-MI are characterized by an excessive stroke risk. Sundboll *et al.* showed the adjusted stroke rate ratio to be increased by about 30-fold. The risk of stroke remained higher during the first year post-MI and in the years to follow.^71^ In such a context, heart rate lowering drugs should be used post-MI to ensure cardioprotection in combination with drugs that have evidence-based efficacy to reduce the risk of stroke.^72,73^

## 4. Conclusions

The latest ESC (2024)^4^ and ESH (2023)^3^ guidelines recommend SBP 120–129 mmHg and DBP 70–79 mmHg in hypertensive patients, including those with CAD. Clinicians should adhere to recommended targets while avoiding antihypertensive treatment de-escalation due to concerns over the J-curve. While a J-curve effect should be considered in the early stage of AMI, available data do not provide definitive DBP thresholds for increased risk. Individualized treatment strategies to manage BP in post-MI remain of key importance to balance BP control with the patient-specific risk profile and comorbidities.

## Data Availability

There are no new data associated with this article.
